# Supplementary material for: Tocotrienols in Different Parts of Wild Hypericum perforatum L. Populations in Poland
Source: Molecules. 2025 Mar 2;30(5):1137. doi: 10.3390/molecules30051137 (PMC11902190; doi:10.3390/molecules30051137)
Supplement: Supplementary file 1 [file molecules-30-01137-s001.zip › molecules-3420715-supplementary.pdf]

Figure S1. *Hypericum perforatum* L. aerial parts.

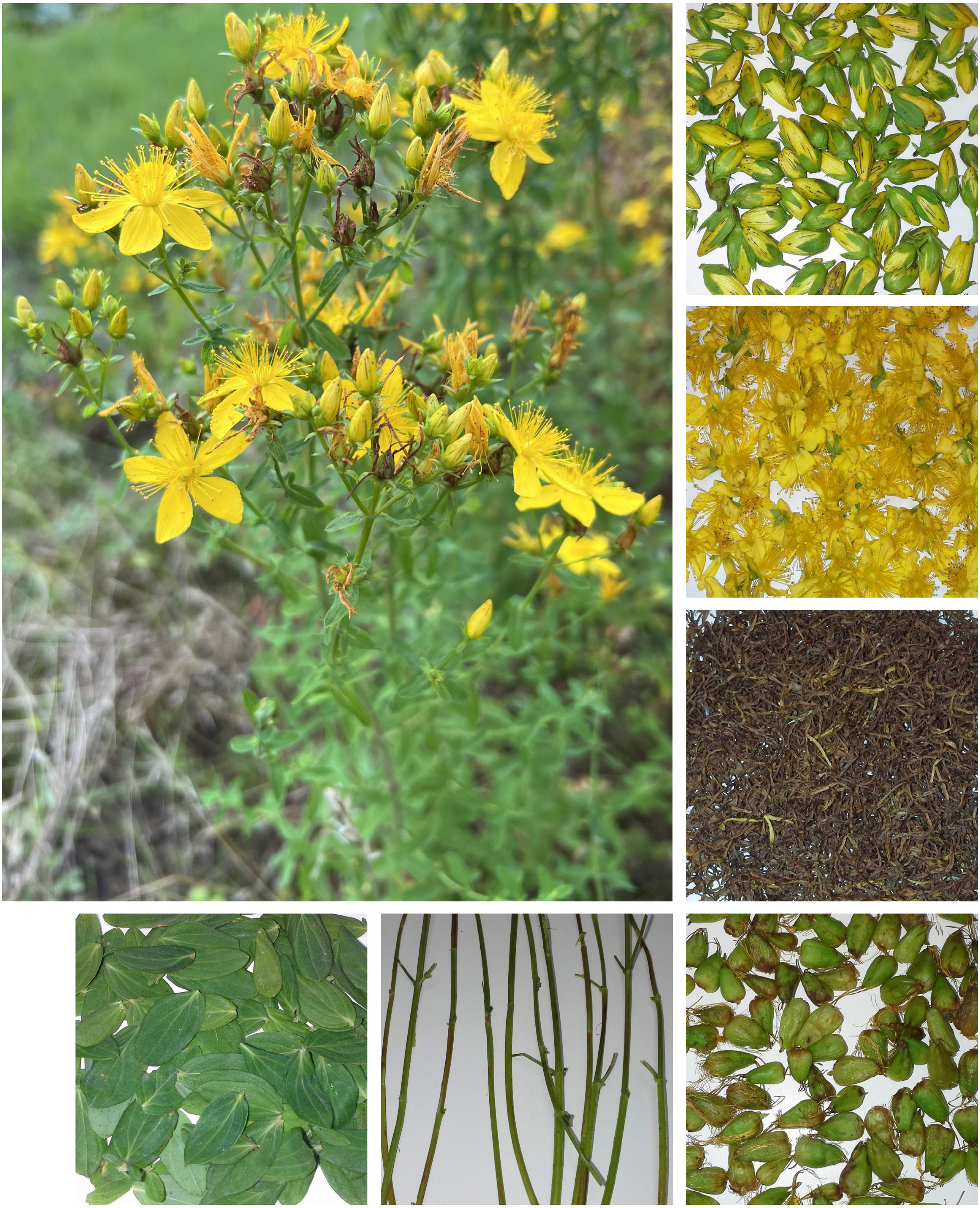

Table S1. Content and ratio of tocopherols and tocotrienols in stems, leaves, flower buds, flowers, dead petals and seed pods of wild *H. perforatum* harvested in Poland.

Plant part	Tocochromanols, mg/100 g dw											Ratio Ts/T3s
	α-T	β-T	γ-T	δ-T	α-T3	β-T3	γ-T3	δ-T3	Total Ts	Total T3s	Total Ts+T3s	
**Stems**												
Min	0.3	tr	tr	—	tr	—	tr	1.0	0.3	1.0	1.3	0.2
Max	6.5	0.5	0.7	—	0.3	—	0.3	4.7	7.7	5.3	12.9	1.5
Average	2.1	0.2	0.2	—	0.1	—	0.1	2.5	2.5	2.8	5.3	0.9
STDEV	1.8	0.2	0.2	—	0.1	—	0.1	1.2	2.1	1.4	3.3	0.4
Coefficient of variation	0.871	1.053	0.999	—	1.301	—	0.600	0.471	0.829	0.489	0.627	0.471
**Leaves**												
Min	17.2	0.2	0.9	tr	0.1	tr	0.1	14.6	29.4	20.5	49.8	1.5
Max	37.0	0.8	2.3	0.3	1.8	tr	0.5	23.5	6.9	2.9	7.9	0.4
Average	27.3	0.4	1.5	0.1	0.4	—	0.3	19.7	18.3	14.7	33.1	0.9
STDEV	6.8	0.2	0.4	0.1	0.5	—	0.1	2.8	40.3	25.8	66.1	2.1
Coefficient of variation	0.249	0.498	0.287	1.094	1.214	—	0.507	0.143	0.236	0.144	0.159	0.279
**Flower buds**												
Min	23.7	1.8	3.2	0.9	17.5	0.5	1.1	29.4	29.6	48.5	78.1	0.6
Max	33.1	4.3	7.1	2.4	29.9	1.1	5.3	37.8	46.8	74.1	120.9	0.1
Average	28.2	3.1	4.7	1.6	22.7	0.8	2.7	33.1	37.6	59.3	96.9	0.5
STDEV	3.4	0.9	1.2	0.5	4.6	0.2	1.4	2.6	5.2	7.9	9.9	0.8
Coefficient of variation	0.122	0.288	0.252	0.322	0.202	0.267	0.531	0.079	0.138	0.134	0.102	0.180
**Flowers**												
Min	20.2	1.9	2.6	1.1	1.9	0.1	0.4	26.6	25.8	29.0	54.7	0.7
Max	29.6	4.3	5.7	2.3	5.3	0.3	1.7	37.8	42.0	45.0	87.0	1.1
Average	24.2	3.0	4.0	1.5	3.7	0.2	0.9	31.1	32.8	35.8	68.6	0.9
STDEV	2.9	0.9	1.0	0.5	1.2	0.1	0.4	4.0	4.6	4.4	6.3	0.2
Coefficient of variation	0.122	0.305	0.258	0.304	0.339	0.421	0.522	0.128	0.139	0.124	0.092	0.180
**Dead petals**												
Min	2.6	0.3	0.7	0.4	0.5	tr	0.1	19.4	4.1	19.9	24.0	0.2
Max	11.7	2.9	2.4	0.9	1.3	tr	0.5	26.7	17.9	28.5	46.4	0.7
Average	6.1	1.5	1.3	0.6	0.7	—	0.2	23.2	9.6	24.1	33.7	0.4
STDEV	3.1	1.1	0.5	0.2	0.3	—	0.2	2.6	4.7	2.9	6.0	0.2
Coefficient of variation	0.503	0.750	0.390	0.261	0.382	—	0.751	0.112	0.488	0.119	0.179	0.477
**Unripe seed pods**												
Min	1.0	tr	12.1	1.2	0.2	—	0.2	29.6	14.3	30.0	44.3	0.4
Max	3.0	0.4	16.7	2.5	1.4	—	0.5	45.1	22.6	47.1	69.7	0.6
Average	1.7	0.2	14.6	1.8	0.8	—	0.3	37.9	18.4	39.0	57.4	0.5
STDEV	0.8	0.2	1.5	0.5	0.5	—	0.1	5.0	2.1	4.9	5.4	0.1
Coefficient of variation	0.454	0.829	0.105	0.291	0.615	—	0.409	0.132	0.115	0.126	0.094	0.176

Average values and standard deviations correspond to nine biological samples of each aerial part of *H. perforatum* (*n* = 9). T, tocopherol; T3, tocotrienol; tr, trace amount (below 0.05 mg/100 g dw); dw, dry weight.
